# Contrasting female mate preferences for red coloration in a fish

**DOI:** 10.1093/cz/zoz052

**Published:** 2019-10-18

**Authors:** Charel Reuland, Brett M Culbert, Alessandro Devigili, Ariel F Kahrl, John L Fitzpatrick

**Affiliations:** 1 Department of Zoology, Stockholm University, Stockholm, SE-10691, Sweden; 2 Department of Integrative Biology, University of Guelph, Guelph, N1G 2W1, Canada

**Keywords:** *Dermogenys collettei*, experience, plasticity, pygmy halfbeak, reproductive status, sexual selection, sperm competition

## Abstract

Understanding how animals select their mates requires knowing the factors that shape mate preferences. Recent theoretical and empirical considerations suggest that female mating status can influence the degree to which a female engages in mate choice, with virgin females predicted to be less choosy than mated females. In this study, we investigated mate choice in both virgin and mated females in the pygmy halfbeak *Dermogenys collettei*. Halfbeaks are small, live-bearing, internally fertilizing freshwater fish that live in mixed-sex groups where females have ample opportunity to engage in mate choice. Using a dichotomous choice assay, we quantified and contrasted in virgin and mated females mate preferences for differences in male body size, beak size, and area of yellow and red coloration. We also examined how mating status influenced the amount of time a female associated with the first male encountered and the relative amount of time a female associated with each male. We demonstrate that mate preferences of female halfbeaks are driven primarily by the size of red coloration present on males. Females showed contrasting preferences based on mating status, with virgin females preferentially associating with drab males whereas mated females preferentially associate with males possessing large areas of red. Contrary to expectations, female mating status did not influence how females associate with the first males encountered or how females biased their association time among males. Although the precise drivers of these effects need further studying, our finding highlights a possible explanation for how variation in male ornamentation can be maintained.

Animals often spend considerable time and energy choosing their mates (Rosenthal 2017). Females are typically the choosier sex, due to their greater investment in a single reproductive event and lower reproductive rates than males (Bateman 1948; Trivers 1972; Andersson 1994). However, female discrimination among potential mates can be costly. Choosy females can face costs in the form of increased search time, loss of energy, and/or increased predation risk, potentially reducing survival or fecundity (Janetos 1980; Reynolds and Gross 1990). Therefore, female mate choice is hypothesized to evolve if males differ in their ability to provide females or their offspring with indirect genetic or direct benefits (Gould et al. 1999), if females are able to detect these differences through male cues, and if the direct or indirect benefits of mating with a specific male offset the costs of being choosy (sensu Rosenthal 2017). In species where females receive no other resource beyond sperm from their male partners (i.e. resource-free mating systems) females can exhibit preferences for male sexual ornamentation (e.g. bright coloration), under the assumption that choosing such ornamented males provides either indirect benefits (good genes), exploits an existing sensory bias in females, or arises through a Fisherian process of runaway selection based on (potentially) arbitrary ornamental traits (Andersson 1994; Prum 2010; Rosenthal 2017). Female mate choice is therefore commonly credited as a powerful selective force driving directional selection on male ornamentation (Andersson 1994; Mead and Arnold 2004). However, within-population plasticity in female mate preferences also appears to play a key role in maintaining population-wide diversity in sexual ornaments (Jennions and Petrie 1997; Badyaev and Qvarnström 2002; Chaine and Lyon 2008).

Variation in female mating preferences can arise through among-female differences in preference functions (termed female preference), or choosiness, the effort invested in mate assessment (Jennions and Petrie 1997). Along with external factors that alter the costs associated with mate choice (e.g., predation risk and conspecific density), there is now considerable evidence that plasticity in female mate choice is influenced by internal factors (reviewed in Jennions and Petrie 1997; Rosenthal 2017). In particular, the expression of female mate choice is expected to depend on female mating experience. Virgin females are hypothesized to be less choosy than recently mated females, as virgin females have not yet secured fertilization of their eggs and should therefore have a lower threshold for accepting mating attempts (Kokko and Mappes 2005). Similarly, female mate choice can also depend on female age and experience, with younger/less experienced females potentially exerting different preferences than older/more experienced females (Coleman et al. 2004; Uetz and Norton 2007; Mautz and Sakaluk 2008; Cotter et al. 2010; Bergman et al. 2011). Yet, while the potential impact of female mating status and experience on mate choice are clearly defined in theory (Jennions and Petrie 2000; Kokko and Mappes 2005; Bleu et al. 2012), surprisingly few studies have tested these predictions experimentally (McNamara et al. 2004; Uetz and Norton 2007; Zeh and Zeh 2007; Bergman et al. 2011; Daniel and Rodd 2016).

In this study, we investigated if female mating status influences female mate choice in the pygmy halfbeak *Dermogenys collettei* (Zenarchopteridae), a small, live-bearing, internally fertilizing fish native to the freshwater bodies of south-east Asia (Meisner 2001; Greven 2010). Halfbeaks are sexually dichromatic (both sexes exhibit coloration, but male coloration is more pronounced, see Figure 1), live in loosely-organized, occasionally shoaling, mixed-sex groups, with both sexes interacting frequently (Greven 2010; Ho et al. 2015). Female pygmy halfbeaks produce large, fully-provisioned eggs (i.e., are lecithotrophic), with no additional nutritional investment of developing embryos following internal fertilization (Reznick et al. 2007; Coleman et al. 2009). The high initial costs of producing fully provisioned eggs in lecithotrophic species are hypothesized to favor the evolution of precopulatory mate choice to allow females to maximize their reproductive investment by selecting high-quality mates (Zeh and Zeh 2000; Pollux et al. 2014). Thus, pygmy halfbeaks represent an excellent model for assessing female mate choice. Male pygmy halfbeaks vary in body size and coloration, displaying red/orange and yellow coloration (primarily on their fins) that have been hypothesized to serve as a sexual ornament in these fish (Coleman et al. 2009). Female halfbeaks produce broods on a near-monthly cycle (Ogden et al. 2019) and are able to store sperm for up to 6 breeding cycles following a single mating (Charel Reuland, personal communication). A single mating event can thus have substantial consequences for a female, and we therefore hypothesize that mating status will influence female mate choice. Specifically, we predicted inexperienced virgins to be less choosy than experienced, previously mated females and to discriminate less based on male ornamentation. We tested this prediction under 2 distinct experimental conditions, where females were presented with a dichotomous choice of males that either differed in body size (large vs. small males) or were size-matched. Contrary to our predictions, mating status did not influence female choosiness. However, our findings reveal differential mate preferences for male sexual ornamentation in inexperienced virgin and experienced mated female pygmy halfbeaks.

**Figure 1.**
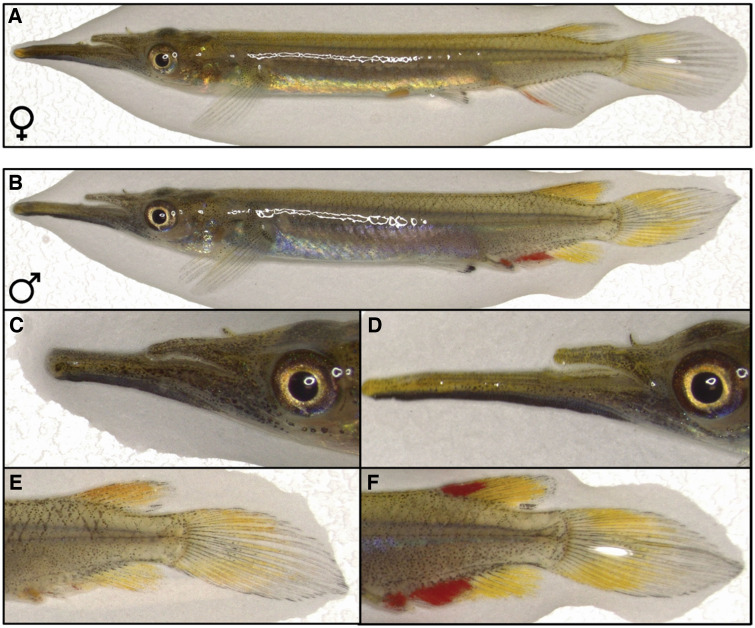
Sexual dimorphism in pygmy halfbeaks and variation in quantified morphological traits of male halfbeaks. (**A**) Full body image of a female pygmy halfbeak with typical fin coloration. Note that female halfbeaks express some coloration on their dorsal, anal, and caudal fin. Abdominal gravid spot is visible. (**B**) Full body image of a male halfbeak with typical fin coloration and beak size. Males have a modified anal-fin, the andropodium, which is used to transfer sperm to females. Males typically exhibit red coloration in the area around the andropodium. Males exhibit variation in beak size, ranging from (**C**) male with small to (**D**) large beaks, and variation in fin coloration, ranging from (**E**) male with small to (**F**) large areas of coloration. Note that coloration close to the anal fin encompasses a small part of the body, the seminal channel, and coloration on this part of the body was included in the analysis. Not shown here is the quantified variation in body size of male fish. See Supplementary Figure S1 for additional details on how body, beak, and coloration areas were measured.

## Materials and Methods

### Study population and rearing conditions

Sexually mature halfbeaks were imported from a commercial supplier (Ruinemans Aquarium B.V., Montfoort, The Netherlands) and housed in mixed-sex groups in aquaria ranging from 72 to 400 L. All tanks were covered with ∼2 cm of gravel, oxygenated, and contained live and artificial plants. Fish were maintained on a 12:12 light:dark cycle at 27°C and fed daily a mixture of ground flake food and freeze-dried *Artemia*. Once per week, fish were additionally fed previously frozen and thawed *Drosophila melanogaster*. To generate virgin females for the experiments described below, gravid females (*n > *80) were isolated in 7.5 L tanks until they produced broods. Once a female had given birth, the female was separated from the offspring (i.e., fry) to prevent maternal infanticide. Fry were housed together in 7.5 L tanks, with up to 7 (the average brood size) fry being housed together in a single tank. When females produced broods of more than 7 fry the broods were split among tanks. Fry were sexed at the initial period of sexual development, around 2-months of age. Males were identified by the thickening of the anal fin at the beginning stages of the development of the andropodium, the modified anal fin used during copulation (Meisner and Burns, 1997, also see Figure 1). After sexing, fry were separated into sex-specific tanks and reared until sexual maturity (∼4 months of age, Greven, 2010; Magyar and Greven, 2007). Tanks were visually separated by opaque barriers, ensuring that fish were naïve to the opposite sex once reaching sexual maturity. Mated females used in this study were sampled from mixed-sex stock tanks maintained in the lab. Therefore, mated females used in this study likely consisted of females at varying stages of their reproductive cycle. Furthermore, mated females were likely on average older than virgin females, although precise ages are unknown for either category. Experiments were approved by Jordbruksverket, the Swedish Board of Agriculture (permit number 2393-2018).

### Dichotomous mate choice assays

**Figure 2.**
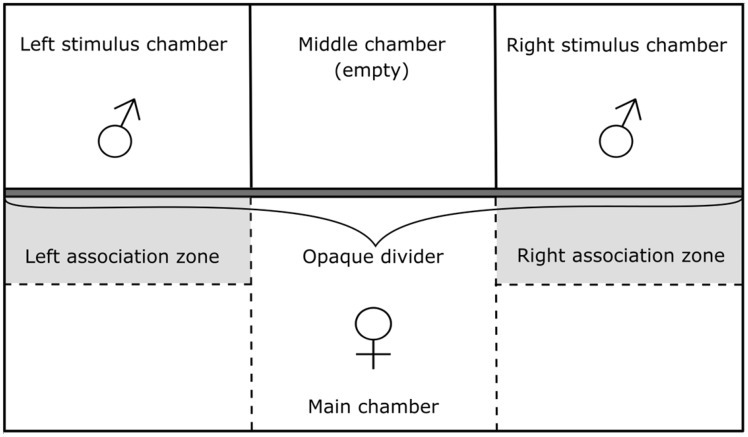
Dichotomous choice chamber used to assess female mate choice. Stimulus males were placed at random in the left and right stimulus chambers and opaque acrylic glass (solid lines) prevented visual contact between the stimulus chambers. During habituation, an opaque divider prevented visual contact between the stimulus males and the chooser female in the main chamber. After habituation, the opaque divider was lifted and the position of the female in either the left, middle, or right side of the tank was noted (here shown by the extended vertical dashed lines). Choice was assessed by recording the time the female spent in either the left or right association zone (15 cm × 5 cm, shown in gray). Association zones as well as divisions of the tank into left, middle, and right compartment were digitally added after recording using landmarks on the wall of the chamber.

Mate choice was assessed in experimental dichotomous choice chamber tanks (45 cm × 25 cm × 20 cm), consisting of one main chamber (45 cm × 15 cm × 20 cm) and 3 smaller, isolated stimulus chambers (each 15 cm × 10 cm × 20 cm) which lay adjacent to each other (Figure 2). The focal female was placed in the main chamber, whereas stimulus males were placed in the left and right stimulus chambers. The middle stimulus chamber was empty (though filled with gravel and water), acting as a spatial separator between the left and right chamber. A transparent glass divider separated the stimulus chambers from the main chamber, allowing the focal females to use visual, but not olfactory, cues to assess stimulus males. Opaque acrylic barriers prevented stimulus males from seeing one another in the stimulus chambers. Opaque dividers surrounded the experimental tank to prevent visual contact of the focal or stimulus fish with the outside environment.

Before each trial, experimental animals were given 60 min to acclimate and familiarize with their environment. During this time, visual contact between the main and stimulus chambers was prevented by an opaque divider. After 60 min, the divider was removed using a pulley system to minimize disturbance and trials were recorded for 60 min using a GoPro Hero 5 Black digital camera (GoPro, Inc., San Mateo, CA, USA), positioned 25 cm above the tank. The duration of time a female spent in each association zone, comprising an area 5 cm in front of the respective stimulus chamber (total dimension: 15 cm × 5 cm, see Figure 2), was used as a measure of female mate preference. The time females spend in such an association zone is a commonly used measure of mating preference that is predictive of female preference during mating interactions (Bischoff et al. 1985; White et al. 2003; Walling et al. 2010). To remove olfactory cues for subsequent trials, all experimental tanks were drained and cleaned after each trial. New gravel (∼1 cm) was then added to the experimental tank, which was then filled with water to a level of 5 cm before every new trial.

Female mate choice is often influenced by male body size as well as female mating status. Therefore, female mate choice was assessed in females that differed in mating status, comparing virgin females versus mated females, under 2 experimental treatments. Females were presented with the dichotomous choice between 2 males matched for body area, henceforth called size-matched (virgin females: *n = *22; mated females: *n = *26) and 2 males that differed in body size (i.e., a large vs. small male, virgin females: *n = *29; mated females: *n = *26). To choose stimulus males in keeping with the desired experimental treatments, pictures of the stimulus males were taken and their body size was measured before the trial (see below). As expected, males in the size-matched treatment did not differ significantly in body size (average absolute difference ± Standard deviation [SD]: 2.14 ± 2.10 mm^2^; 2-sample *t*-test assuming equal variance: *t* = −0.71, *df =* 94, *P = *0.48), whereas the body sizes of the large males were significantly larger than the small males in the treatment where body size differed between stimulus males (average absolute difference *±* SD: 36.80 ± 16.86mm^2^; 2-sample *t*-test assuming unequal variance: *t* = −11.90, *df =* 70.55, *P < *0.001). After being photographed males were rested for 12–96 h before entering a trial. No females or males were reused for any experimental treatments. Males in both experimental treatments were strictly sorted according to body size, with beak and coloration size varying at random.

Females can only make an informed mate choice decision after having visited the association zones of both stimulus males. Therefore, association times for experiments involving informed choice were only included after the female had visited both males’ association zones. In total, 116 trials were conducted and scored for up to 30 min after the female visited both stimulus males. In a minority of cases, females never visited the second male, or visited him late into the video, resulting in less than 30 min of scored video material (*n = *38). Of those 38 trials, 10 were omitted as females never visited the second stimulus male (*n = *7) or informed mate choice was recorded for less than 10 min (*n = *3). Furthermore, trials were immediately terminated if females showed any behavioral irregularities (e.g., repeatedly swimming along the walls of the tank, *n = *3). This resulted in a final sample size of 103 trials being included in the statistical analysis (average body area ± SD: 86.23 ± 20.98 mm^2^; range 57.82–201.16 mm^2^; average standard length ± SD: 30.21 ± 3.16 mm; range 23.84–44.78 mm).

To account for possible side biases of the females, the position of large and small males in either the left or right stimulus chamber was randomized. The positions of the females at the beginning of the trials did not differ between the left, middle, or right side of the tank in both experimental treatments (χ^2^ test, size-matched: χ^2^ = 1.30, *df =* 2, *P = *0.52; large vs. small males: χ^2^ = 4.21, *df =* 2, *P = *0.12). Females did not spend significantly more time in the left or right association zones (paired *t*-test, size-matched: *t =* −1.20, *df =* 47, *P = *0.24, large vs. small males: *t =* −0.27, *df =* 54, *P = *0.78), confirming that females did not express an innate side bias in this experimental setup.

### Measuring morphological traits

Following the behavioral trials, males were sedated in a benzocaine solution and morphological traits were recorded. An image of the fish lying on its left lateral side was captured against a white background (including a scale) using a Leica S9i stereo microscope (Leica Microsystems Ltd., Heerbrugg, Switzerland) and LAS X software (Leica Microsystems Ltd., Heerbrugg, Switzerland). Four male traits possibly involved in female mate choice were analyzed: body size, beak size, and yellow and red coloration area on or close to the fins (Figure 1). Body size and beak size are expected to advertise male competitive ability during intraspecific competition, as male halfbeaks interlock their beaks and “wrestle” until a winner is determined during agonistic interactions (Berten and Greven, 1991). Halfbeaks also exhibit male-biased sexual dichromatism, with males exhibiting more pronounced yellow and red coloration than the comparably less colored females (Figure 1), suggesting a role of coloration in female mate choice (Coleman et al. 2009).

Body and color area measurements were obtained using the polygon selections tool in ImageJ v1.51k (Schneider et al. 2012). Commonly, body size in fish is quantified as the standard length, the distance from the tip of the snout to the caudal peduncle. However, in halfbeaks, the beak is elongated (Figure 1) and including the beak when measuring body size leads to an exaggerated standard length measure. Furthermore, as males use their beak during intrasexual contest competition, we were interested in examining female preference for males based on both body size and beak size. Therefore, we measured both body and beak size as measurements of area. However, other size metrics, including standard length, were explored in alternative models that provided similar conclusions to the model presented in the main text (see Supplementary Table S1). Body size was measured as area by tracing around the fish, omitting all fins (Supplementary Figure S1). Total beak size was measured by tracing the outline of the jaws up to the anterior part of the eye (Supplementary Figure S1). The anterior point of the eye was used as a reference point as it could be more reliably identified than the posterior end of the jaw. To quantify the amount of coloration on the anal and dorsal fins (including adjacent parts of the body in cases where coloration extended from the anal fin), red and yellow color areas were analyzed using a color thresholding method embedded in the ImageJ program. Color thresholding offers a standardized way to analyze color patterns, especially more complex patterns that are either patchy or show a gradient in color intensity. The outlines of the fins were traced with a polygon selection tool and the outside of the fins was cleared. Using the “threshold color” function, areas of coloration were automatically assessed (Supplementary Figure S1). Due to slight changes in the lighting setup used, thresholding values were adjusted slightly between treatments (Supplementary Figure S1). To verify the accuracy of the color thresholding method, a subsample of the images was analyzed both by tracing the color areas of the anal and dorsal fins by hand and by thresholding the image. Color areas obtained using the threshold method and manual tracing values were highly correlated (yellow coloration: *r*^2^ = 0.92, *P < *0.001; red coloration: *r*^2^ = 0.90, *P < *0.001). Therefore, we manually traced color patches on the caudal fin of fishes when glare on the fin hampered automatic recognition of the patterns using color thresholding.

### Statistical analyses

**Figure 3.**
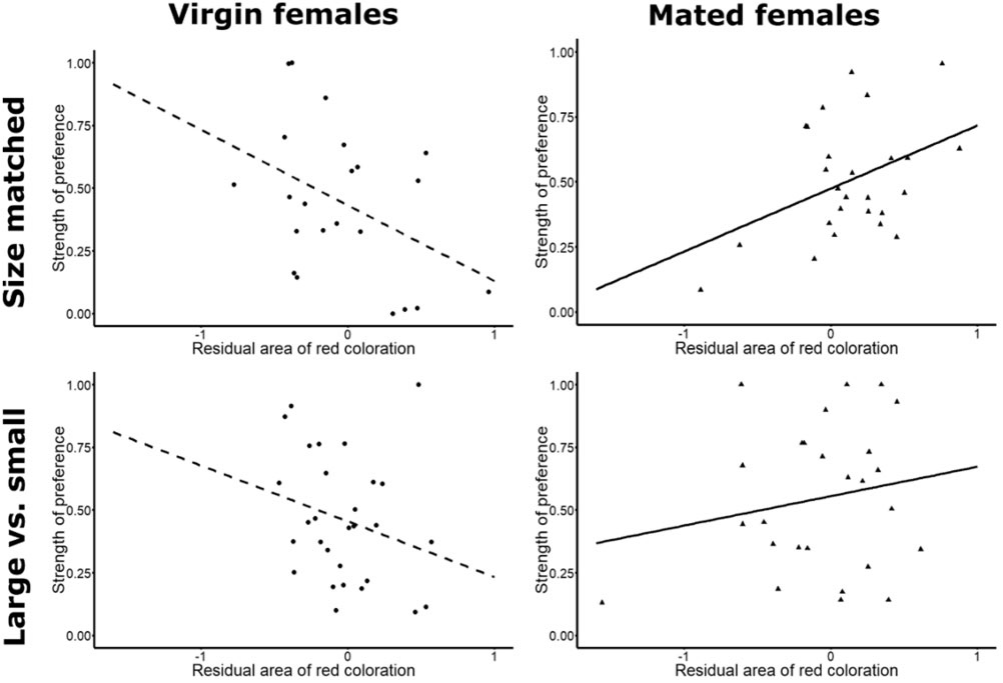
Relationship between the strength of preference of females for the left male and the area of red coloration of the left male in comparison to the right male. *Y*-axis: strength of preference of females for the left male. *X*-axis: residuals for the difference between areas of red coloration of left and right male. Residuals were obtained by constructing a linear model with the difference of red coloration of the left and right male as the dependent variable and the differences between body size, yellow coloration area, and beak size, as well as female mating status and experimental treatment as predictor variables (*n = *103). For visual representation of the dataset, we divided plots according to female mating status and experimental treatment. **Top**: (**left**) virgin (*n = *22, *r* = −0.42) and (**right**) mated (*n = *26, *r *=* *0.41) females choosing between 2 size-matched males. **Bottom**: (**left**) virgin (*n = *29, *r* = −0.25) and (**right**) mated (*n = *26, *r *=* *0.19) females choosing between a large versus small male. Note that separated plots and indicated Pearson’s *r*-values are for visualization only and that the final statistical analysis presented in the “Results” section was conducted on a linear model encompassing both reproductive states and experimental treatments (*n = *103).

In our experimental setup, females were given a binary choice between 2 males. Therefore, females could choose males based on morphological traits alone or based on how different these traits are between stimulus males. To account for the dependence of data, association of a female with a male was calculated as strength of preference score for the left male (an arbitrary male). The strength of preference for the left male was calculated as follows: the time that a female spent in left males’ association zone/the time that a female spent in left and right males’ association zones. As such, strength of preference scores ranged from 0 to 1, with values above 0.5 indicating that the female spent more time in the left association zone and more time in the right association zone if the strength of preference score is below 0.5. To assess the morphological variation between the left and right stimulus males, we calculated the differences (in body size, beak size, total red fin coloration area, and total yellow fin coloration area) between left and right males (measurements of morphology of the right male were subtracted from measurements of the left male). To determine if female mate choice varied in response to morphological traits of the males, we constructed linear models with the “strength of preference for left male” scores as the dependent variable. Female reproductive status (virgin vs. mated) was added as a fixed effect, the difference between body size, beak size, red area and yellow area of the 2 stimulus males were treated as covariates, and all possible interactions between female reproductive status and morphological covariates were included in the model. Two separate models were run at first for the 2 experimental treatments (“size-matched” and “large vs. small” males). However, as findings were comparable between the 2 models (see Figure 3 and Supplementary Table S2), size-matched and large versus small male treatments were ultimately combined into a single model that included the 2 treatments as a fixed effect, including all possible interactions between fixed effects and covariates (*n* trials = 103).

The time the female spent with the first male she encountered in the experimental setup, up until she explored the tank further and enters the association zone of the second male (the beginning of a possible informed choice) was recorded and analyzed. We measured this variable as a possible proxy for a female’s motivation to mate, as virgin females should theoretically be more motivated to mate compared with previously mated females that scored fertilization of their eggs. Hence, virgin females are predicted to stay with the first male they encounter for longer durations. Furthermore, females may stay longer with the first male based on their morphological traits (e.g., body size or coloration). As this analysis focused solely on the time spent with the first male, 10 trials where females only briefly (<10 min) or never visited the second males’ association zones were included (4 trials with size-matched stimulus males and 6 trials with large and small males, with half the trials in each treatment featuring a virgin or a mated chooser female). Data were analyzed using a linear model with the time that the female spent in the first male’s association zone as the dependent variable and morphological traits of this male, female reproductive status, experimental treatment, and their interaction term as predictor variables. To improve the fit of the model residuals, the dependent variable was transformed using a standardized Yeo-Johnson transformation (λ = 0.19, mean = 10.04, SD = 3.70) (Yeo and Johnson, 2000) included in the VGAM package (Yee, 2010).

Lastly, we evaluated a female’s association bias (the relative time spent with the preferred male compared with the unpreferred male; calculated as the time in the male association zone the female was for the longest divided by the time in both association zones). Differential choosiness of virgin and mated females (with virgin females being less choosy), as is predicted by theory (Jennions and Petrie 2000; Kokko and Mappes 2005; Bleu et al. 2012), should result in nonchoosy females (e.g., virgins) associating indiscriminately with 2 males, thus having low-association biases. As such, we interpret association bias as a metric to assess female choosiness. Scores around 0.5 indicate no or weak association bias (low choosiness) and scores near 1.0 indicate a strong preference for one specific male (strong choosiness). We accounted for possible influences of male traits by including in the statistical model the differences between male morphological traits (calculated as the measurements of morphology of the male, the female spent the longest time with minus the measurements of morphology of the other male). Data were analyzed using a linear model with the female association bias as the dependent variable and differences between male morphological traits, as well as female reproductive status, experimental treatment, and their interaction terms as predictor variables.

All analyses were completed using R version 3.4.5 (R Core Team 2017). In all models, 3-way interactions were excluded to facilitate model interpretability and interaction terms with *P *>* *0.1 were dropped from the final model. Significant effects were obtained using the “Anova” function included in the “car” package (Fox and Weisberg 2011). The final model fit was assessed through visual inspection of the residuals.

## Results

### Female preferences for male traits

We detected a significant interaction effect between the area of male red coloration and female mating status, with virgin females associating less and mated females associating more with males possessing large areas of red (Figure 3, Table 1). In contrast, female pygmy halfbeaks did not preferentially associate with males based on differences in male body size, size of the beak, or area of yellow coloration (Table 1). The mating status-specific responses to red coloration persisted between the experimental treatments (Table 1). Virgin and mated females preferred to associate with drab (i.e., less red) or colorful (i.e., more red) males, respectively, regardless of the difference in size between the males (Figure 3). The lack of a treatment effect in our model was surprising, particularly for the large versus small treatment, as one may expect larger animals to possess larger areas of red. However, in our trials, larger body size was not synonymous with larger red areas (Supplementary Table S3).

**Table 1.** Female strength of preference for male traits

Predictor variable	*t*	*P*
Male body size	−1.47	0.14
Male beak size	1.14	0.26
Total area of male red coloration	1.64	0.86
Total area of male yellow coloration	0.67	0.50
Female mating status	−1.26	0.15
Treatment	0.82	0.41
Total area of male red coloration: Female mating status	−2.93	**0.004**

Testing for the influence of male traits, female mating status, and experimental treatment on female strength of preference. Effects were tested in a model with strength of preference as the dependent variable and morphological differences between stimulus males, as well as female reproductive status and experimental treatment as predictor variables (*n = *103). *P*-values in bold indicate significant effects.

### Mating status effects on female motivation and choosiness

Female association time with the first male they encountered in the trial, a proxy for their motivation to mate, was not influenced by female mating status or experimental treatment (Table 2). There was a statistical trend for the area of male red coloration, suggesting male red coloration possibly having influenced the time a female spent with the first male encountered and females staying longer with redder males. Other male traits did not significantly influence female association time with the first male.

**Table 2.** Testing for influence of male traits, experimental treatment, and female mating status on the female’s motivation to associate with the first male encountered and the relative amount of time a female associated with the preferred male compared with the other male, expressed as an association bias score

	Predictor variable	*t*	*P*
Female association time with first male encountered (s) (*n =* 113)			
	Male body size	−0.35	0.73
	Male beak size	0.30	0.76
	Total area of male red coloration	1.72	0.09
	Total area of male yellow coloration	1.19	0.24
	Female mating status	0.82	0.41
	Treatment	0.10	0.92
Female association bias score (*n =* 103)			
	Male body size	1.46	0.15
	Male beak size	−1.45	0.15
	Total area of male red coloration	−0.39	0.70
	Total area of male yellow coloration	−0.94	0.35
	Female mating status	0.51	0.61
	Treatment	1.09	0.28

The relative time the female allocated between the 2 males, measured as an association bias score, can be seen as a proxy for the choosiness of a female. In our experiment, neither female mating status nor experimental treatment explained the differential strength of choice in female halfbeaks (Table 2). Furthermore, association biases scored were not significantly influenced by the magnitude of trait differences between male traits.

## Discussion

We demonstrate that female mating status influences the expression of female mate preferences for male ornamentation in the pygmy halfbeak. Specifically, our results show that females alter their behavior in response to variation in the amount of red coloration present on males. However, the direction of female preference differs between mated and virgin females. Mated females spent more time associating with males with relatively greater areas of red coloration and less time with males expressing relatively small areas of red coloration. This result is in accordance with the idea that females exert precopulatory mate choice for more colorful males, a finding that matches female mate choice decisions in numerous sexually dichromatic species (Andersson 1994; Rosenthal 2017). However, contrary to our expectations, virgin females preferred to associate with males possessing *smaller* areas of red coloration. These results were found across our experimental treatments. When presented with the dichotomous choice of either size-matched or large versus small males, mated and virgin females always differed in their preference for red coloration, and mated/virgin females displayed the same preferences for male coloration in both treatments. The opposing direction of mate preference between mated and virgin females suggests that female mating status may play a role in the maintenance of male phenotypic variation in pygmy halfbeaks.

The mating status-dependent expression of female mating preference we uncovered in pygmy halfbeaks is surprising. Mated female preference for red coloration appears to fit within the standard sexual selection paradigm where females use sexual ornaments expressed in males to either secure indirect fitness benefits via the acquisition of “good genes,” to produce “sexy sons,” or due to pre-existing sensory biases (Andersson 1994; Rosenthal 2017), although confirmation of these processes in halfbeaks remains outstanding. But why do virgin females prefer males with less red coloration? As virgin females lack sperm from previous matings, they may benefit relatively more compared with already mated females to mate with males with high ejaculate quality and sperm number. Virgin females may, therefore, prefer drab males if they have superior ejaculate quality, even if low coloration is indicative of perhaps otherwise low indirect genetic benefits. Sexual selection theory broadly predicts trade-offs between energetically costly traits (like ornaments) and ejaculate quantity or quality (Simmons et al. 2017). For example, in the red-backed fairy-wren *Malurus melanocephalus* breeding males with less exaggerated ornaments produce denser ejaculates (Rowe et al. 2010), whereas Evans (2010) reported a negative genetic correlation between iridescent coloration and sperm swimming speed and viability in guppies *Poecilia reticulata*. In contrast, a recent meta-analysis found limited evidence that male morphological ornamentation is positively related to ejaculate quality (Mautz et al. 2013). However, uncovering if differential mate choice by virgin females is adaptive requires a greater understanding of how sexual conflict operates and the phenotypic and genetic patterns of covariance in sexual traits in halfbeaks.

An additional, nonadaptive explanation for our results could be that virgin females, who were housed in sex-specific tanks and naïve to males prior to the experiment, were sexually inexperienced and therefore react differently from older and more experienced mated females when assessing male sexual ornaments. Exposure to sexual signals and social experience (or the lack thereof) are well known to influence mate preferences and various effects have been described (Dion et al. 2019). Virgin females, for example, did not have the opportunity to observe experienced females choosing males, which could potentially shape mate choices of inexperienced females (Dugatkin and Godin 1992; Rosenthal 2017), or observe male–male interactions that could be important if male sexual ornamentation predicts social dominance and influences mate choice (Wong and Candolin 2005). Although rearing females in sex-specific tanks is a common practice to generate virgins for experimental purposes in many species (Houde 1987; Jones et al. 2000; Evans and Magurran 2001; Fedina and Lewis 2007), the potentially deprived social environment these females experienced could have influenced subsequent mate choice decisions. Moreover, experience, age, and mating status typically covary in females, and each of these factors has the potential to influence female responses to male sexual displays. For example, in the satin bowerbird *Ptilonorhynchus violaceus*, younger (presumably less experienced) females have lower tolerance for intense courtship displays by males compared with older females (Coleman et al. 2004; Patricelli et al. 2004). Whether female halfbeaks exhibit a similar experience-dependent response to male ornaments remains unclear. An interesting avenue for future investigation would, therefore, be to disentangle the effects of female mating status, age, and experience on mate choice in halfbeaks. More specifically, raising virgins either with or without visual but never physical access to males/mixed-sex groups may help in understanding whether mating status or experience drove the observed effect. Furthermore, testing whether virgin or inexperienced females are less adept at spotting members of the opposite sex, or prefer to associate with same-sex conspecifics, may help in understanding how mate choice decisions are generally shaped. In our experiment, male halfbeaks could not engage in elaborate courtship displays that require the male to be under the female or circle around her (see Greven 2010). Future work should, therefore, explore the link between male ornamentation and courtship behavior and its impact on choosing behavior of virgin and mated females.

We found no evidence that mated or virgin females differed in their motivation or choosiness. This result was unexpected, as there is ample evidence that virgin females and mated females can differ in choosiness, with virgin females being comparably less choosy (Uetz and Norton 2007; Zeh and Zeh 2007; Bergman et al. 2011; Daniel and Rodd 2016; but see McNamara et al. 2004). However, adaptive choosiness based on mating status is not thought to be expressed in every species. Adaptive choosiness may be difficult to evolve in a species if the costs of establishing such a mechanism are high. In species with first mate sperm precedence, for example, virgins may not have lowered mating thresholds, because virgins that mated indiscriminately at first would be unable to “undo” any mistake of mating with a bad quality male by simply remating at a later time with a male of higher quality (Kokko and Mappes 2005). High direct costs of mating should also facilitate the evolution of strong choosiness (Bleu et al. 2012), and therefore hinder the emergence of females with low choosiness. Another explanation for the observed lack of difference in choosiness between virgin and mated female pygmy halfbeaks might be that there is a little opportunity (or benefit) for adaptive choosiness to evolve in this species. Pygmy halfbeaks form large mixed-sex groups and male pygmy halfbeaks frequently court females and attempt to mate with them (Greven 2010; Ho et al. 2015). As such, it may be that receptive virgin females do not face problems finding a mate in this species and pygmy halfbeaks could, therefore, be falling under the theoretically predicted thresholds by Kokko and Mappes (2005) where adaptive choosiness should evolve. Sperm precedence effects and relative costs of mating are great candidate traits to examine in the pygmy halfbeak and to compare between species with or without adaptive choosiness in order to empirically test the aforementioned predictions. Finally, female choosiness may be expressed in a more subtle or complex way in pygmy halfbeaks, which may not have been detected using the metrics measured in this study. The time the female spent with the first male she encountered in the experimental setup for example, which we interpreted as motivation to mate, may be influenced by individual behavioral variation, including the boldness of an individual and their innate tendency of exploration (Sih et al. 2004).

The role of male coloration as an ornament involved in female choice was previously suggested for halfbeaks (Coleman et al. 2009). In our study, we experimentally confirmed that area of red coloration is indeed a trait of choice for female halfbeaks, but we did not find any effect for area of yellow coloration. In comparison to area of red coloration, area of yellow coloration showed overall little variation, and this may therefore explain why no choice based on yellow coloration was found (see description in Supplementary Figure S1). Furthermore, females may show stronger reactions to saturation and intensity of male yellow coloration, rather than area of coloration, as was measured in this study. Yellow coloration could also perhaps be used by males to contrast and enhance the regions of red coloration, while being in and of itself not attractive to females, an effect that has been described for black coloration in the guppy *P. reticulata* (Brooks, 1996). We, therefore, interpret the nonsignificant result of yellow coloration with caution until the effect of color intensity has been examined.

Females did not associate differently based on visual assessment of the males’ beak and body size. Weapon and body size are common proxies of social status in other species (Emlen 2008), but this relationship has not yet been validated in the pygmy halfbeak. As such, this study by design cannot infer if females may or may not discriminate based on male social status. However, this study provides valuable insight into candidate traits and could demonstrate that if female pygmy halfbeaks adjust mating decisions based on a male social status, they cannot do so by visually discriminating between beak (weapon) and body size. Instead, females would have to resort to other methods of assessment, either using other traits as a proxy, by directly observing fights between males, or by assessing body size through other senses such as their lateral line sensory organs. The role of male social status and the perception of male traits, like odor cues, through other, nonvisual, sensory organs is an interesting topic for future investigations of female mate choice in this species.

Our study demonstrates female mate choice in the pygmy halfbeak and identifies one visual male trait involved. Surprisingly, virgin and mated females showed contrasting preferences for red coloration. These results suggest that female mating status, or factors closely associated with it like experience, can influence the perceived attractiveness of male traits. Our finding highlights the necessity to test mate choice on females of both mating statuses in order to understand mate choice in its entirety (a criticism previously raised by Kokko and Mappes 2005). Lastly, our findings imply that contrasting preferences of virgins/inexperienced and mated/experienced females may lead to disruptive selection on male traits in the pygmy halfbeak. This finding may, therefore, offer an alternative explanation to how variation in male ornamentation can be maintained in a population, and we encourage future research to test the prevalence of this effect, as well as its impact on ornament evolution, in other study systems.

## Supplementary Material

zoz052_Supplementary_Data
