# Supplementary material for: Contrasting female mate preferences for red coloration in a fish
Source: Curr Zool. 2019 Oct 18;66(4):425–33. doi: 10.1093/cz/zoz052 (PMC7319460; doi:10.1093/cz/zoz052)
Supplement: zoz052_Supplementary_Data [file zoz052_supplementary_data.zip › zoz052-Suppl_Data/Supplementary Material.docx]

**Supplementary Material**

**Contrasting female mate preferences for red coloration in a fish**

Charel Reuland^a,*^, Brett M. Culbert^b^, Alessandro Devigili^a^, Ariel F. Kahrl^a^, and John L. Fitzpatrick^a^

^a^Department of Zoology, Stockholm University, Stockholm, Sweden SE-10691;^b^Department of Integrative Biology, University of Guelph, Guelph, Canada N1G 2W1

*Address correspondence to Charel Reuland. Email: [charel.reuland@zoologi.su.se](mailto:charel.reuland@zoologi.su.se)

Handling editor:  Ingo Schlupp

Received on 5 June 2019; accepted on 12 October 2019

**Table S1:** Female strength of preference for male traits with different body and beak size measurements. Testing for influence of male traits, female mating status and experimental treatment on female strength of preference. Effects were tested in models with strength of preference as the dependent variable and morphological differences between stimulus males, as well as female reproductive status as predictor variables. **(a)** Male body size measured as standard body length and beak size as beak length. **(b)** Body size measured as eye to caudal peduncle length (omitting the beak) and beak size as beak length. **(c)** Body size measured as body area from eye to caudal peduncle (omitting the beak) and beak size as beak area. The results are qualitatively similar to those obtained from models using body area and beak area, which is presented in Table 1. Boldface p-values indicate significant effects.

**Table S1:**

|  | **Predictor variable** | **t** | **P** |
| --- | --- | --- | --- |
| (a) Standard body length, Beak length | |  |  |
|  | Male standard body length | -0.82 | 0.42 |
|  | Male beak length | 0.43 | 0.67 |
|  | Total area of male red coloration | 1.57 | 0.88 |
|  | Total area of male yellow coloration | 0.70 | 0.49 |
|  | Female mating status | -1.26 | 0.15 |
|  | Treatment | 0.95 | 0.35 |
|  | Total area of male red coloration: Female mating status | -2.77 | **0.007** |
|  |  |  |  |
| (b) Eye to caudal peduncle body length, Beak length | |  |  |
|  | Male body length eye to caudal peduncle | -0.82 | 0.42 |
|  | Male beak length | 0.15 | 0.88 |
|  | Total area of male red coloration | 1.57 | 0.88 |
|  | Total area of male yellow coloration | 0.70 | 0.49 |
|  | Female mating status | -1.26 | 0.15 |
|  | Treatment | 0.95 | 0.35 |
|  | Total area of male red coloration: Female mating status | -2.77 | **0.007** |
|  |  |  |  |
| (c) Eye to caudal peduncle body area, Beak area | |  |  |
|  | Male body area eye to caudal peduncle | -1.47 | 0.14 |
|  | Male beak area | 1.09 | 0.28 |
|  | Total area of male red coloration | 1.64 | 0.86 |
|  | Total area of male yellow coloration | 0.67 | 0.50 |
|  | Female mating status | -1.26 | 0.15 |
|  | Treatment | 0.82 | 0.41 |
|  | Total area of male red coloration: Female mating status | -2.93 | **0.004** |

**Table S2:** Female strength of preference for male traits in the size-matched and large vs. small male treatments. Testing for influence of male traits and female mating status on female strength of preference. Effects were tested in two models with strength of preference as the dependent variable and morphological differences between stimulus males, as well as female reproductive status as predictor variables. **(a)** Females choosing between two size-matched males. **(b)** Females choosing between a large and small male. Boldface p-values indicate significant effects.

| **Predictor variable** | | **t** | **p** |
| --- | --- | --- | --- |
| (a) Female strength of preference for male traits – size-matched treatment (*n =* 48) | | | |
|  | Male body size | -1.53 | 0.13 |
|  | Male beak size | -0.80 | 0.43 |
|  | Total area of male red coloration | 1.53 | 0.54 |
|  | Total area of male yellow coloration | -0.86 | 0.49 |
|  | Female mating status | -0.34 | 0.34 |
|  | Total area of male red coloration: Female mating status | -2.67 | **0.01** |
|  | Total area of male yellow coloration: Female mating status | 2.03 | **0.05** |
|  |  |  |  |
| (b) Female strength of preference for male traits – large vs. small male treatment (*n =* 55) | | | |
|  | Male body size | -1.69 | 0.10 |
|  | Male beak size | 1.73 | 0.09 |
|  | Total area of male red coloration | 0.99 | 0.95 |
|  | Total area of male yellow coloration | -0.004 | 1.00 |
|  | Female mating status | -1.00 | 0.32 |
|  | Total area of male red coloration: Female mating status | -1.88 | 0.07 |

**Table S3:** Correlations between male traits. A correlation matrix based on Spearman’s rank correlation coefficients is presented for correlations between male body size, male beak size, total area of male red coloration and total area of male yellow coloration. **(a)** Correlations between male traits of both treatments. **(b)** Correlations between male traits in the size-matched treatment. **(c)** Correlations between male traits in the large vs. small treatment. Significance levels are indicated in superscript where * indicates p ≤ 0.05, ** indicates p ≤ 0.01 , and *** indicates p ≤ 0.001.

| (a) Correlations between male traits – all treatments (*n =* 206) | | | |
| --- | --- | --- | --- |
|  | **Body size** | **Beak size** | **Area of red** |
| Body size | - |  |  |
| Beak size | 0.91^***^ | - |  |
| Area of red | -0.10 | -0.11 | - |
| Area of yellow | 0.55^***^ | 0.50^**^ | 0.11 |
|  |  |  |  |
| (b) Correlations between male traits – size-matched treatment (*n =* 96) | | | |
|  | **Body size** | **Beak size** | **Area of red** |
| Body size | - |  |  |
| Beak size | 0.93^***^ | - |  |
| Area of red | -0.04 | -0.08 | - |
| Area of yellow | 0.36^***^ | 0.30^***^ | 0.12 |
|  |  |  |  |
| (c) Correlations between male traits – large vs. small treatment (*n =* 110) | | | |
|  | **Body size** | **Beak size** | **Area of red** |
| Body size | - |  |  |
| Beak size | 0.89^***^ | - |  |
| Area of red | -0.15 | -0.12 | - |
| Area of yellow | 0.61^***^ | 0.61^***^ | 0.13 |

**
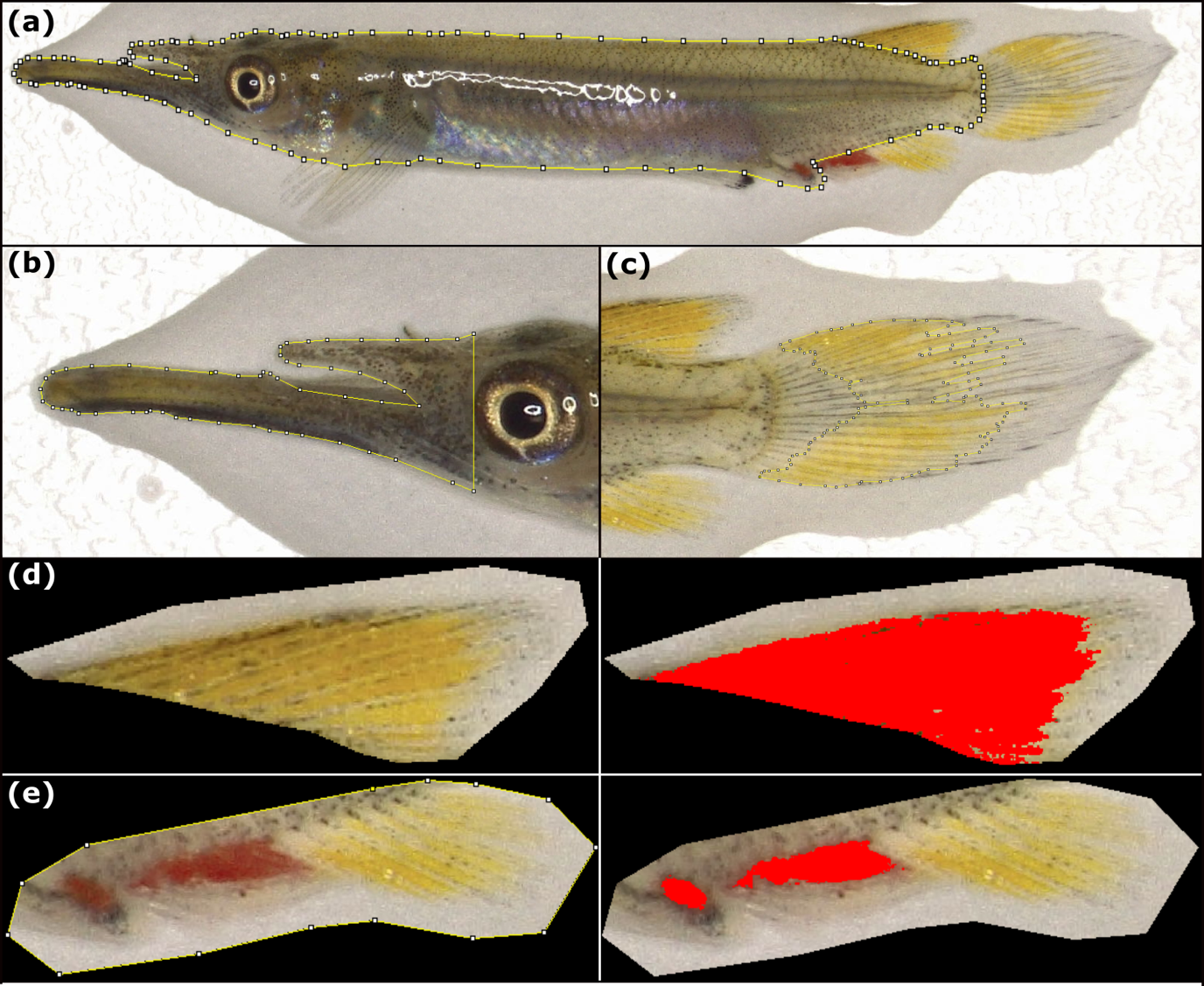
**

**Figure S1:** Quantification of morphological features (body size, beak size, red coloration area, yellow coloration area). All measurements were taken in ImageJ v1.51k (Schneider et al. 2012). Average body size of males measured was 86.23 ± 20.98 mm^2^ and mean differences in body size between two stimulus males in a trial were 20.65 ± 21.31 mm^2^. Beak size of male halfbeaks was on average 7.83 ± 2.09 mm^2^, with areas of beak differing on average 1.95 ± 1.89 mm^2^ between males in a trial. Average total area of red coloration measured was 0.40 ± 0.33 mm^2^ (mean difference 0.35 ± 0.26 mm^2^) and average total area of yellow coloration was 8.34 ± 3.60 mm^2^ (mean difference 3.62 ± 2.82 mm^2^). **(a)** Body size. The outline of the body was traced using a polygon selection tool. Note that all fins, the inside of the mouth, as well as the fleshy part of the andropodium were omitted when assessing body size. **(b)** Beak size. The outline of the beak and the jaws was traced using a polygon selection tool. If the fish had their mouth open (pictured), the inside of the mouth was not traced and the silhouette followed the outline of the lips of the fish. The anterior point of the eye was used as a reference point for the posterior end of the beak area as it could be more reliably identified than the posterior end of the jaw itself. **(c)** Yellow (and red) coloration on the caudal fin. Color areas on the caudal fin were traced by hand using a polygon selection tool. Yellow areas were traced along any visible discoloration of the fin, disregarding any differences in intensity or saturation of these color areas. Red areas on the caudal fin were generally rare and if present, small and lightly “sprinkled” on the fin. If present, all red areas were traced using a polygon selection tool and summed up. **(d)** Yellow coloration on the anal and dorsal fin (pictured) quantified using a thresholding protocol. The outlier of the fins was roughly traced with a polygon selection tool and the outside of the fins was cleared (left image). Using the “threshold color” function areas of coloration were automatically assessed. Yellow on the anal fin was assessed with values Hue: 19-40 (Pass checked), Saturation 110 – 255 and Brightness 150-255. Yellow on the dorsal fin was assessed with values Hue: 19-40 (Pass checked), Saturation 110 – 255 and Brightness 120-255. Brightness valued differed for anal and dorsal fin due to slight differences in background coloration, possibly due to different thicknesses, of these fins. Due to slight changes in the lighting setup used, thresholding values were adjusted accordingly in between treatments. Notably, saturation values were adjusted ± 5 units and brightness values ± 10 units around the protocols given in (d) and (e). **(e)** Red coloration on the dorsal and anal fin (pictured) quantified using a thresholding protocol. Red on the anal and dorsal fin was assessed with values Hue: 0-18 and 240-255 (Pass unchecked), Saturation 145 – 255 and Brightness 80-255.
